# Stagewise Overview of Issues Influencing Organizational Technology Adoption and Use

**DOI:** 10.3389/fpsyg.2021.630145

**Published:** 2021-03-17

**Authors:** Mina Saghafian, Karin Laumann, Martin Rasmussen Skogstad

**Affiliations:** Department of Psychology, Norwegian University of Science and Technology, Trondheim, Norway

**Keywords:** organizational change, human-technology interaction, organizational intervention, technology acceptance, technology adoption

## Abstract

This paper provides a stagewise overview of the important issues that play a role in technology adoption and use in organizations. In the current literature, there is a lack of consistency and clarity about the different stages of the technology adoption process, the important issues at each stage, and the differentiation between antecedents, after-effects, enablers, and barriers to technology adoption. This paper collected the relevant issues in technology adoption and use, mentioned dispersedly and under various terminologies, in the recent literature. The qualitative literature review was followed by thematic analysis of the data. The resulting themes were organized into a thematic map depicting three stages of the technology adoption process: pre-change, change, and post-change. The relevant themes and subthemes at each stage were identified and their significance discussed. The themes at each stage are antecedents to the next stage. All the themes of the pre-change and change stages are neutral, but the way they are managed and executed makes them enablers or barriers in effect. The thematic map is a continuous cycle where every round of technology adoption provides input for the subsequent rounds. Based on how themes have been addressed and executed in practice, they can either enhance or impair the subsequent technology adoption. This thematic map can be used as a qualitative framework by academics and practitioners in the field to evaluate technological changes.

## Introduction

The topic of organizational change has received a lot of attention in the past years. There are various kinds of changes and transformations that organizations go through. Organizational changes could be due to merging and acquisition, or cultural, structural, procedural, and technological changes (Smith, 2002). Organizational change management is about taking action toward a defined state through practices that are different from the routine practices (Burnes, 2009; Maali et al., 2020) to reach a goal. The goal of technology change is to offer better products and services through replacing the existing with the new technology (Aremu et al., 2019). The extent to which an organization is able to adapt to required changes is referred to as organizational readiness. With regard to technology changes, readiness encompasses technological infrastructure, knowledge, (human) resources, and managerial commitment to change, and the extent of readiness is a precursor to successful organizational change management (Han et al., 2020; Maali et al., 2020). Therefore, each of the factors that contribute to readiness deserves attention.

Throughout history, there have been three eras of technological advancement, namely the agricultural, industrial, and digital eras (Cascio and Montealegre, 2016). The base of the digital era's technologies is information and communication technologies (Cascio and Montealegre, 2016) that are further divided into three groups: Function IT, which enhances task execution and precision; Network IT, which enhances communication; and Enterprise IT, which redesigns and standardizes workflow. A new era is the fourth industrial revolution, or Industry 4.0, which marks the evolution of technology from mechanical to electrical to digital, and now to even more automation and intelligent networking of systems through smart manufacturing (Lewis and Naden, 2018; Sony and Naik, 2020). Technology adoption and implementation processes in Industry 4.0 are more complex than traditional technology implementations because they involve a wider range of connected technologies, calling for work process readjustments, industrial scale changes, and changes in the society on a global level (Lewis and Naden, 2018). The pace of technological evolution is exponential, and the nature of technologies is more disruptive (Molino et al., 2020) than previous technological eras. This implies that a number of subprojects need to be coordinated and managed strategically (Rojko, 2017). Industry 4.0 has become a topic of great interest among scholars and practitioners since 2013 (Culot et al., 2020). With the rapid advancement of technology, various disciplines and sectors have been influenced by technological evolution. We need to know what we have learned from the past eras of technological advancements to be able to deal with the challenges of the present and future eras. Therefore, an overview of the important factors and their role in technology adoption success is needed. A number of models and theories have been introduced and used in the existing literature. However, they present their findings through different theoretical lenses and they differ in their area of focus and scope. They point to different and sometimes overlapping and rather general factors of the technology adoption process. There is no one overview of all these factors that are mentioned dispersedly in the literature. It is not clear when these factors play a role and when they act as enablers or barriers to technology adoption. Therefore, the following questions arise:

1. What are the main themes (issues/factors) in organizational technology adoption?
2. When do the themes play a role and at what stage of the technology adoption process?
3. How do the themes exert an influence as enablers or barriers?

This paper aims to consolidate different theoretical frameworks, findings, and suggested interventions into one stagewise overview. This will help to emphasize timing in the process, making it easy to create subprojects that can be delegated. Having such an overview will enable us to consolidate new findings from new technological revolutions into the map, updating it with new trends, and to customize it to the internal and external organizational environment. Therefore, it is both general and customizable when applied per industry or organization. The overview will organize these issues into stages, clarifying their role as antecedents or after-effects, enablers, or barriers. The key actors are identified when applicable, and the recommended practices for a better technology adoption process are highlighted. This will help identify future research needs and foci.

In the next section, an overview of the main theoretical frameworks used in past studies is introduced, and an overview of previous findings about influential factors in technology adoption is provided.

### Theoretical Background

The current literature has used a number of theories and models for studies related to information systems (IS) and information technology communication (ICT) (Eze et al., 2019). A description of each of them is beyond the scope of this paper, but the most prominent models of technology adoption and change management theory are presented in this section to give an idea of the lenses through which the most prominent factors in technology adoption have been identified to date.

#### Technology Adoption Models

The models most referred to can be grouped into two categories of individual level and organizational/firm level models. At the individual level, the theory of reasoned action (TRA) by Ajzen and Fishbein (1980) posits that a person's behavior, through intention, is influenced by their attitude and their perceived subjective norms about that behavior. Attitude is shaped by the personal belief and view of the behavior, while the subjective norms is about how other's evaluations are perceived and form motivation to engage in a behavior (for a more detailed overview, see Dwivedi et al., 2012). This model was adopted from the field of psychology into IS models (Dwivedi et al., 2012). The model was improved into the theory of planned behavior (TPB) by Ajzen in 1985, and builds on TRA by including perceived behavioral control (PBC), which is important when actions are not under full volitional control. “According to the theory of planned behavior, perceived behavioral control, together with behavioral intention, can be used directly to predict behavioral achievement” (Ajzen, 1985, p. 184).

Another widely used model is the technology acceptance model (TAM) developed by Davis (1989). It has been considered a “powerful tool to represent and understand the determinants of users' IT-acceptance process” (Dwivedi et al., 2012, p. 167). This model posits that intention to use technology is affected by attitude, which in turn influences perceived usefulness (PU) and perceived ease of use (PEOU). PU is based on the belief as to whether the application or innovation helps people perform their job better and helps them improve task performance. PEOU refers to the evaluation of whether or not using the application is worth the effort (Dwivedi et al., 2012). PU is also influenced by PEOU (Dwivedi et al., 2012).

An improvement was made to TAM by incorporating more consumer-related factors (Slade et al., 2015), leading to the unified theory of acceptance and use of technology (UTAUT) by Venkatesh et al. in 2003 in acknowledgment that the determinants of acceptance of technology vary across models. The authors studied some of the main models in the IT field to create UTAUT. It postulates that acceptance and behavior are directly determined by performance expectancy, effort expectancy, social influence, and facilitating conditions. The effect of the core constructs is moderated by age and gender, experience, and volition of use (Venkatesh et al., 2003). Both TAM and UTAUT have been criticized for not capturing the complexity of technology adoption adequately, for being too focused on individuals' beliefs and perceptions, and for having reached a plateau of how much they can contribute to the field of technology adoption (Shachak et al., 2019), which is rapidly evolving and expanding.

Diffusion of innovation (DoI), developed by Rogers (1995), focuses on both individual and firm level technology adoption. It integrates three major components: adopter characteristics, innovation characteristics, and the innovation decision process. In the innovation decision step, there are five steps, namely knowledge, persuasion, decision, implementation, and confirmation, that are influenced by communication channels among the members of a similar social system over a period of time. Perceived characteristics of innovation include five main constructs—relative advantage, compatibility, complexity, trialability, and observability—that have been proposed as factors affecting any innovation acceptance. In the adopter characteristics step, five categories are defined: early adopters, innovators, laggards, late majority, and early majority (for more details, see Baker, 2012 in Dwivedi et al., 2012). Therefore, this model takes into account not only the features of technology, but also individual characteristics and organizational processes. This model is consistent with another firm level model, the technology, organization and environment framework (TOE) developed by Tornatzky and Fleischer (1990). However, the TOE also incorporated the environmental context into the model, which is an advantage over the DoI (Oliveira and Martins, 2011). This model tries to show how technology innovation decision-making is affected by the technological context (technology characteristics and availability), the organizational context (formal and informal linking structure, communication, size, and slack), and the environmental context (market and industry structure, technology support and infrastructure, government regulations) (for more details, see Baker, 2012). The technological context is concerned with the equipment and practices internal to the organization as well as externally available technologies, and draws attention to the disruptiveness of new technology and adoption risks (Baker, 2012). The organizational context is about the organizational structure, management, resources, and processes, and how they affect innovation adoption in organizations. The environmental context is the arena in which the organization operates (Oliveira and Martins, 2011) and how factors such as governmental policies regarding safety or efficiency, industrial maturity and its effect on the strategy for innovation, and the presence and capacity of technology providers influence technology adoption and implementation (Baker, 2012). While DoI is more about the antecedents and determinants of technology adoption, TOE is more about the contextual factors (Oliveira and Martins, 2011).

These models are often used in the literature, especially in the IS, management, and health sectors. However, regarding technology adoption, TRA and TPB do not account for many factors relating to the technology itself and there is no clear indication of the different stages of the adoption process involved. TAM and UTAUT are at the individual level of adoption, while DoI and TOE are applied at the organizational level (Oliveira and Martins, 2011). They deal with many organizational structure factors and contextual factors (Oliveira and Martins, 2011), but there is not enough emphasis on interventions such as training and participation that require the more active engagement of users and leaders in the change process. The next section provides an overview of some of the change management models that could highlight the role of leadership and intervention.

#### Organizational Change Management Theories

Technology adoption is not a passive process: it requires active leadership engagement. Therefore, it is also important to look at some of the prominent models of change management. “Change management is the process of continually renewing an organization's direction, structure, and capabilities to serve the ever-changing needs of external and internal stakeholders” (Moran and Brightman, 2000, p. 66). One of the oldest models of change management is the three-step “unfreeze,” “change,” and “refreeze” model of change introduced by Lewin (1947) (Cummings et al., 2016). This approach was further elaborated by Schein and was regarded as a cognitive redefinition, learning to think in a new way (Schein, 1999). The first step (unfreezing) involves creating motivation and readiness for change by creating a balance between perceived threats of change and the perceived psychological safety to embrace change (Schein, 1996; Wirth, 2004). The second step (change) involves the identification of what needs to be changed and what should be the outcome, and it can benefit from observing role models that have achieved the desired state, or mentors, as well as learning through trial and error and thus scanning the possible solutions to enhance learning and coping with ongoing change (Schein, 1996; Wirth, 2004). The third step (refreezing) involves habituations and maintenance of the new behaviors consistent with the change through the development of a new identity and relationships within the broader cultural context (Wirth, 2004).

Kotter (1996) introduced a model of change implementation involving eight steps: (1) create a sense of urgency; (2) create a guiding coalition; (3) create a vision and strategy; (4) communicate the change vision; (5) empower broad-based action; (6) generate short-term wins; (7) consolidate gains and further changes; and (8) anchor change into the culture (for more detail, see Kotter, 1996; Appelbaum et al., 2012). Armenakis and Harris (2009) developed a model focused on creating change readiness. The model consists of five components: (1) discrepancy, which is about depicting a difference between the current state and the potential future state; (2) efficacy, which is about creating trust in the ability to accomplish the change goals; (3) appropriateness, which is concerned with being convinced that the change solution is the best solution; (4) principal support, which is about the provision of support during the change; and (5) personal valence, which addresses the perceived personal benefit in the change process. In order to reinforce the five components, seven strategies are mentioned. These strategies are management of information, persuasive communication, formalization of activities, human resource practices, diffusion practices, rites and ceremonies, and active participation (Armenakis and Harris, 2009; Sætren and Laumann, 2017).

Cummings and Worley (2015) presented a five-step model for effective change management including the following steps: (1) motivating employees for change; (2) creating a vision to clarify the why and what of the change; (3) providing political support for change and preventing individuals and groups from blocking the change; (4) managing the transition through planning of activities and maintaining commitment; and (5) sustaining momentum through resource management, change agents, support systems, and skill development (Cummings and Worley, 2015).

There are certain overlaps between these models, but they are not specific to technology and innovation management and interventions. Nevertheless, incorporating the theories about how to manage change into an overview of different perspectives on technology adoption and use at the firm level could create a more comprehensive overview of the entire process of technology introduction and use, from preparing for technology change to maintaining technology use. Many of these frameworks have been used in the literature. An overview of the recent findings is presented next.

### Existing Literature

There have been numerous studies on the topic of technology adoption and implementation. A review of the important factors in technology adoption in Industry 4.0 highlighted strategic management and goal setting, employees, the digitalization and harmonization of technology, change management, and cyber security management (Sony and Naik, 2020). There was no clear stagewise division, but the importance of timing was indicated by emphasizing implementation in a timely manner. With regard to time, having a realistic timeframe that accounts for the unpredictable obstacles during the process was noted, and the need to adjust workloads at a given time was indicated (Maali et al., 2020).

Most research has stemmed from IS and ICT (Eze et al., 2019) in accordance with the third era of technological revolution (Cascio and Montealegre, 2016). A number of facilitators and barriers are proposed. Literature reviews have shown that organizational culture, strategy, and resources management should be aligned to minimize obstacles to adoption (Kumar et al., 2018; Mohtaramzadeh et al., 2018; Shin and Shin, 2020). However, the obstacles are not clearly distinguished and the mechanism of adjusting the culture and strategy is rather abstract. In other studies, internal upper management support was found to a prominent factor (Chung and Choi, 2018; Lu et al., 2018; Mohtaramzadeh et al., 2018; Maali et al., 2020; Ukobitz, 2020). Furthermore, awareness of the state of affairs in the organization's external environment was found to be essential (Mohtaramzadeh et al., 2018; Vaishnavi et al., 2019). Organizational structure was found to impact technology adoption (for more detail, see Aremu et al., 2018). Other studies have pointed out the importance of change agents and influential and senior users (Keyworth et al., 2018; Molino et al., 2020) in forming normative pressure and influencing the perceptions of other users (De Benedictis et al., 2020). Communicating the reasons for change and provision of training were also mentioned as important facilitators of technology adoption (Maali et al., 2020; Molino et al., 2020). The importance of focusing on the users and users' beliefs as a facilitator of adoption (Mohammed et al., 2018) was mentioned in the literature review by El Hamdi and Abouabdellah (2018). Other studies have suggested that technical issues and technical maturity can be barriers to successful technology change (Keyworth et al., 2018). The importance of a work environment that is supportive to the employees, the participation of skilled key actors, and fostering cross-functional teamwork were mentioned in the study by Kumar et al. (2018). The study by Eze et al. (2019), identified the major factors of adoption as technology's ease of use, a focus on the users, and management. Eze et al. (2019) pointed out that the process of technology adoption is a dynamic process. None of the stages is static, and therefore the different factors involved can influence the adoption process at different stages (Eze et al., 2019). In addition to the aforementioned factors and the dynamic nature of the technology adoption process as posited by Eze et al. (2019), it is important to highlight that when people engage with technology and interact with it, they adapt to it in different ways, showing different outcomes of the use (Schmitz et al., 2016) and application of technology. This is even more complex in today's sociotechnical systems (Lewis and Naden, 2018).

Most of the recent work and reviews show failure in organizational technology adoption and low technology usage (Decker et al., 2012; Sligo et al., 2017). The existing reviews mostly consider the same models of technology adoption embedded in IS field (Hwang et al., 2016; Koul and Eydgahi, 2017; Lai, 2017). Fewer studies have focused on firm level technology adoption (Oliveira and Martins, 2011) than on individual technology adoption. In the existing models and theories on how to manage technology change, the terms “antecedent” and “after-effects” are not clearly distinguished. The studies on antecedents of success and failure are inconsistent (Decker et al., 2012). Therefore, what can be considered as antecedent to success can also be antecedent to failure, which implies these antecedents can be both enablers of success and barriers to it. According to Decker et al. (2012), these antecedents are rooted in different theoretical foundations and can include cultural factors, decision-making processes, and power hierarchy, leadership, and past change failures. Other reviews have offered a detailed account of barriers (Kruse et al., 2016), but it is not clear to which stage of change they belong. The stages of change are not always clearly defined in the reviews, and therefore it is difficult to consolidate the findings from different reviews into a chronological and consistent overview to help identify antecedents and after-effects, enablers, and barriers. Furthermore, the prominent models and theories either target restricted aspects and stages in the technology adoption process with respect to the interest of that specific discipline and without capturing the broader organizational ecosystem, or they are too broad and do not pinpoint the specific issues at particular stages that one needs to be aware of while studying technological adoption processes. The desired outcome is mostly the displayed usage behavior. Usage does not show what users actually think about the new technology, how it was implemented, and how they will incorporate that into future technology rounds. These theories imply a linear process, while Industry 4.0 highlights a need for constant change that is more complex than a mere linear process. Furthermore, the change theories present guidelines and advice for managers on how to implement change from a managerial perspective, and the experience of technology adopters and the broader organizational ecosystem is not clearly stated. Nevertheless, the models have important components that can be integrated into one general overview accounting for both individual level and firm level adoption. It is important to map the existing findings about the technology adoption process in order to be able to evaluate the process and practices against the emerging challenges of technology adoption and its complexities, as characterized by the new technological era (Oliveira and Martins, 2011; Culot et al., 2020). This will help identify new areas of research about where and when improvements are needed and how can they be implemented.

There is a wide range of theoretical frameworks and issues to consider when introducing new technologies in organizations. As such, the motivations for this research and for a qualitative approach were: (1) the lack of one comprehensive overview in the literature of all the issues and themes at different stages of organizational technology adoption; (2) the lack of clarity of when and how these issues acts as enablers and barriers, antecedents and after-effects, and the inconsistent use of terminology; and (3) the broad scope of the research question about a complex system and the need to identify and map the issues mentioned in the literature can be better explored through a qualitative approach to literature review. This literature review aimed to identify and map the scope of the existing literature and it was based on systematic review protocol (Munn et al., 2018). The map can be used for further detailed investigations and systematic literature reviews (Bramer et al., 2017). The aim is to present a stagewise thematic map of important themes and issues to consider from pre-change to post-change, with consideration for future technology change rounds. The map covers issues ranging from selection to acceptance, adoption, and implementation by the stakeholders involved. It is transferable across disciplines. It can be used as a tool to analyze how the relevant themes might influence the technology adoption process by acting as enablers or barriers, and how themes in one stage become antecedents to the themes in the subsequent stage.

In the following section, the materials and method used for the literature search are presented, followed by the data analysis. The results and discussion of the themes, and a thematic map thereof, are presented after that, followed by a general discussion and the conclusion of the paper.

## Method

The data were collected through a search of literature in recent publications that addressed themes relating to organizational technology acceptance and adoption. Qualitative analysis was chosen due to the explorative nature of the research question. Qualitative analysis in this case provides an overview of all the themes that should be investigated when adopting a new technology. This paper is a literature review based on qualitative and quantitative research. It attempts to map and identify the main themes and to provide information that can be used by researchers and practitioners seeking to gain an overview of contemporary technological adoption issues in organizations.

### Literature Search

The literature review was carried out based on qualitative research methods in order to ascertain which issues (qualitative) or factors (quantitative) influence technology adoption and use in organizations. A Boolean search was conducted in databases containing electronic journals, including ISI Web of Science and PsycINFO. A combination of search terms was used, including:

Organizational change AND (technology adoption/ technology readiness/ technology acceptance/ technology endorsement/ embrace/ approval/ reception/ maturity/ willingness/ undertaking)

The truncated forms of these terms were also searched. Truncation was applied by using the asterisk function, and an exact phrase search was conducted using the double quote character. ISI Web of Science and PsycINFO were chosen because they have peer reviewed publications from various fields and mainly technology and social sciences, which would be most suitable for the topic of the paper. ISI Web of Science provided relevant articles, selected by humans rather than robots, from disciplines that were closely related to the topic of the research presented in a wide range of journals. PsycINFO provided the psychological and organizational psychology point of view for the development of the thematic map. This will also enhance transferability, as more disciplines and research fields are represented in the thematic map. Furthermore, they allow for conducting a Boolean search, which is quite helpful in using different search terms. The search period was limited to publications from 2013 to the end of 2020, the reason being that this period saw the start of publications on Industry 4.0 adoption (Culot et al., 2020). However, additional sources beyond this time period were used to elaborate on the main concepts and definitions of the themes and subthemes that resulted from an analysis of the selected articles.

**Figure 1:**
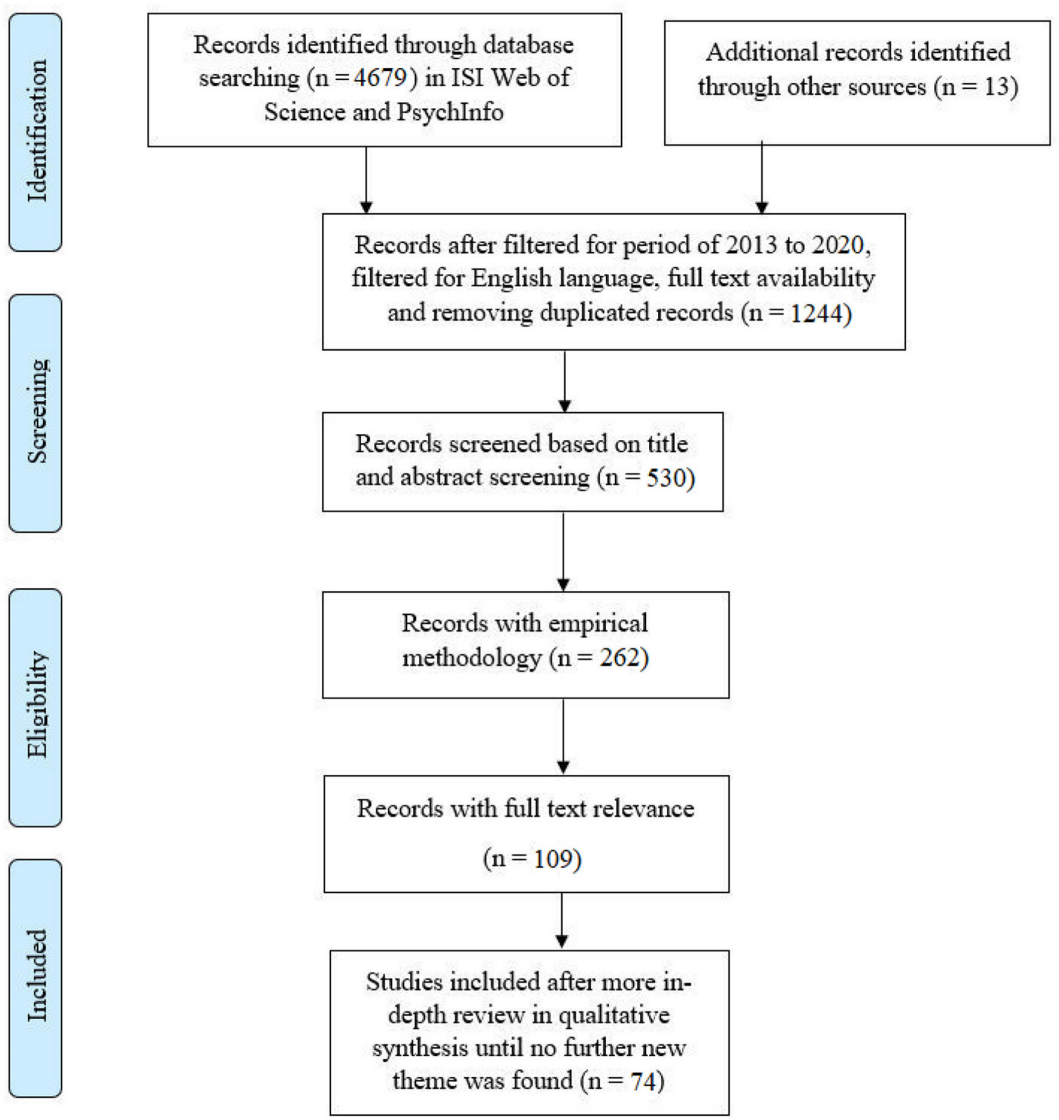
Overview of the steps taken to conduct data collection and the criteria for inclusion of sources to be analyzed.

Conducting the review started with developing the search terms, then entering them in the ISI Web of Science first, retrieving the results, filtering for the years 2013–2020, filtering for English language and full text availability, and transferring the remaining publications to the library. The same procedure was applied to the PsycINFO database. The resulting records were added to the library. Duplicated records were continually removed from the library as new sources were added over the period of data collection; the removed duplicates were not recorded. The publications were screened by title and abstract relevance where articles indicated or implied factors and issues affecting technology use in organizations, including tangible and intangible technologies. Next, the selected articles were filtered for empirical methodology. Theoretical papers were not considered. In order to embed the research in evidence, publications that used qualitative and quantitative methods to obtain their results empirically were chosen as opposed to theoretical papers where we are not certain to what extent they have been verified. This adds to the credibility and trustworthiness of the data used to develop the thematic map. The result was 262 peer-reviewed articles. They were read in full and evaluated for relevance to the topic of the review (issues affecting technology use and adoption); if the article introduced an issue or factor that was found to be important or related to technology uptake in the organizational setting, then it was included in the in-depth analysis. The resulting papers were imported to NVivo 12. Critical reading of articles and evaluation of fit were conducted (Savin-Baden and Major, 2013). This resulted in 109 articles. At this stage, data analysis started in the form of coding. This process is explained in further detail below. However, it is important to clarify that data analysis included 74 articles before establishing saturation. See Figure 1 for an overview of the process inspired by the PRISMA model (Moher et al., 2009) to show the data collection process.

While saturation is generally viewed as “a criterion for discontinuing data collection/or analysis” (Saunders et al., 2017, p. 1894), there are different approaches to it. In this paper, saturation was reached when new papers did not lead to the emergence of a new code or theme, which is consistent with the inductive thematic saturation model (Saunders et al., 2017). This is when the data only feed into the existing codes and do not result into any new codes (Urquhart, 2013), new themes (Given, 2016), or new theoretical insights (Saunders et al., 2017). In this paper, the articles considered for data analysis were categorized primarily according to the database from which they were collected, starting with ISI Web of Science, which has a larger collection and is more multidisciplinary. When the articles ceased to reveal a new theory, thematic map, or concept that was not already covered in previous publications or whose constituent elements had not already been addressed, saturation was assumed. This was reached after 74 articles had been analyzed. A further three articles were added from the references to explain the themes and subthemes in more detail.

It must be acknowledged that it is not possible to establish absolute saturation: it should not be viewed as an absolute point that the researcher reaches, but rather a process in which the researchers establish a degree of saturation that they consider to be enough, knowing that it is always possible to have new and emerging codes and themes (Strauss and Corbin, 1998). However, if adding new data ceases to contribute anything new, data collection becomes redundant (Strauss and Corbin, 1998; Saunders et al., 2017). There is always a possibility that certain information regarding different themes and subthemes, different dimensions of a code, and disconfirming evidence derived from the data may have been excluded. According to Saunders et al. (2017, p. 1903), “an uncertain predictive claim is made about the nature of data yet to be collected.” There is a large volume of publications on this broad research topic, and one can never claim that all the publications that could have some sort of relevance have been reviewed or included.

### Data Analysis

In this paper, thematic analysis was conducted because of the advantages of this method. “Through its theoretical freedom, thematic analysis provides a flexible and useful research tool, which can potentially provide a rich and detailed, yet complex, account of data” (Braun and Clarke, 2006, p. 78). This method allows for “identifying, analyzing and reporting patterns (themes) within data. It minimally organizes and describes your data set in (rich) detail” (Braun and Clarke, 2006, p. 79). Braun and Clarke (2006, p. 82) posit that “a theme captures something important about the data in relation to the research question and represents some level of patterned response or meaning within the data set.” The importance of the theme or “the ‘keyness’ of a theme is not necessarily dependent on quantifiable measures … but rather on whether it captures something important in relation to the overall research question” (Braun and Clarke, 2006, p. 82).

Conducting thematic analysis is best done through the six phases of thematic analysis as indicated by Braun and Clarke (2006), which can overlap with other methods of qualitative research such as grounded theory. This phasic approach to conducting thematic analysis is merely a guideline and not a strict protocol and rules about conducting analysis. However, it provides a systematic and iterative approach. In phase one (familiarizing oneself with the data), the publications were screened and later thoroughly read to ensure prolonged engagement with the data set. In the second phase (generating initial codes), initial ideas about what the smallest meaningful unit of text could be, and the pattern observed in the text across different publications, were captured through codes (Braun and Clarke, 2006). Open coding was done on the texts in a systematic way, and the codes were constantly reviewed and adjusted throughout the process with other research team members to ensure trustworthiness. Codes were categorized into groups in phase three (searching for themes), and different groupings and hierarchical structures were reviewed to make sure that the resulting structure was consistent and captured the different codes derived from the data. This was reviewed and adjusted in collaboration with other research team members in an iterative process in accordance with phase four (reviewing themes). The identified themes and subthemes were reviewed for naming and grouping in accordance with phase five (defining and naming themes), and the results were reported in phase six (producing report). For more details, see (Bowen et al., 1989).

**Figure 2:**
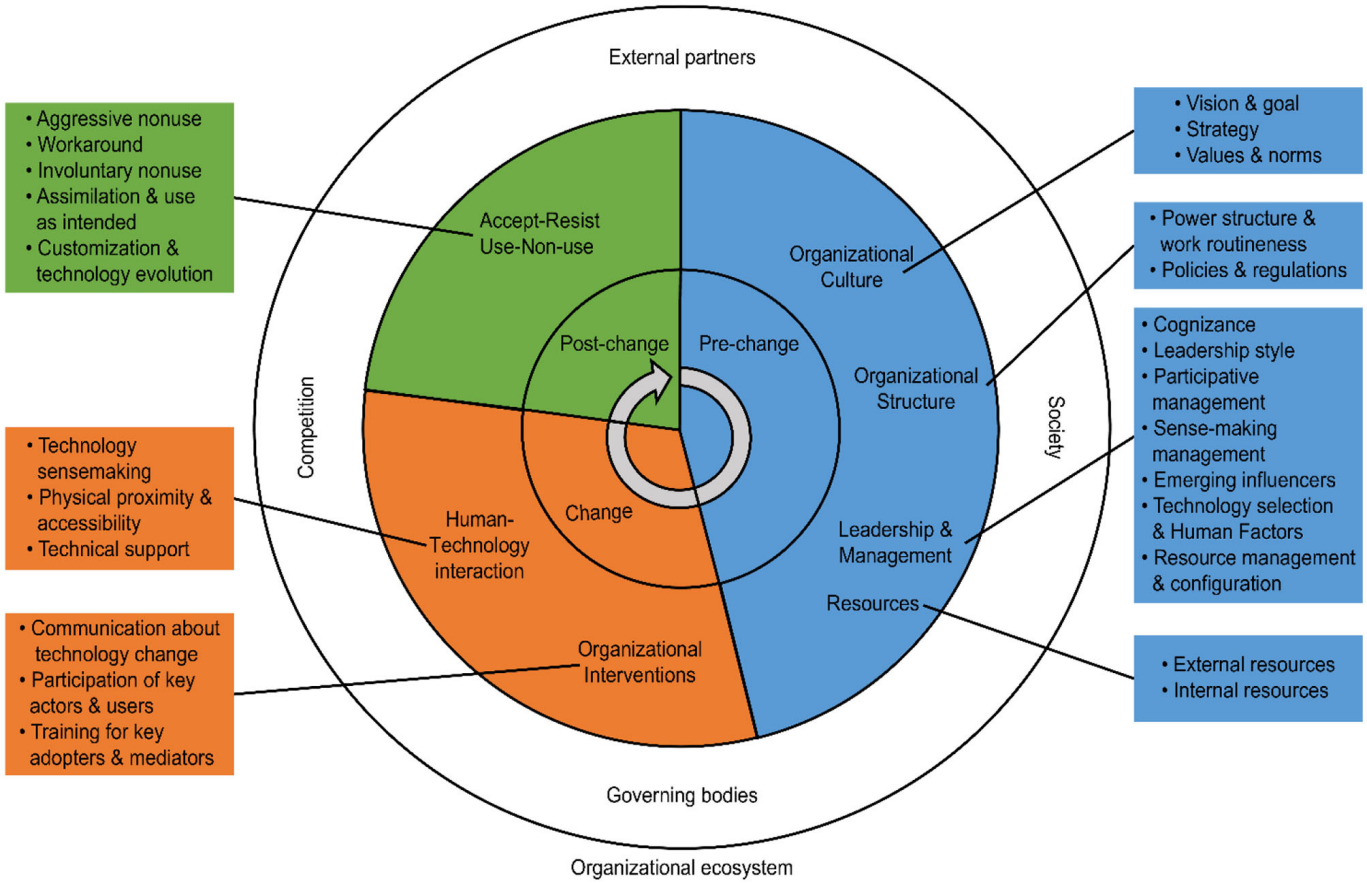
Thematic map of organizational technology selection, adoption, and intervention themes in the pre-change, change process, and post-change stages.

Thematic analysis has different approaches. In this paper, inductive thematic analysis was chosen, where the codes and the themes are developed from the data. Semantic coding was initially applied. “With a semantic approach, the themes are identified within the explicit or surface meanings of the data, and the analyst is not looking for anything beyond what a participant has said or what has been written” (Braun and Clarke, 2006, p. 84). In the next step, latent coding was performed to identify the more subtle and latent meanings and patterns. Analysis at the latent level “starts to identify or examine the underlying ideas, assumptions and conceptualizations … and ideologies … that are theorized as shaping or informing the semantic content of the data” (Braun and Clarke, 2006, p. 84). Latent coding uncovers the overlapping themes and the various terminologies assigned to the same concepts. For example, in the article by Parris et al. (2016), they state that “Market demands and threats posed by competitors are primary stimuli for firms to explore innovation as [a] means to change.” This unit of text can be coded to the theme of external environment, as it reflects how an organization will be influenced to adopt a change by its environment. The themes and subthemes were then organized into a thematic map to show an overview of “the relationship between codes, between themes, and between different levels of themes” (Braun and Clarke, 2006, p. 20). Through this map, our view of the order of the main themes and subthemes in the different stages of technology adoption and implementation is depicted (see Figure 2). In order to provide an overview of the data used for analysis, a descriptive account of the data with regard to the percentage of the references representing each sector, region, and method used was also prepared.

This research took place within the post-positivist paradigm, which posits that reality exists, but it is difficult or impossible to determine whether true reality has been found: it is “imperfectly apprehendable” (Guba and Lincoln, 1994, p. 110). In this paradigm, the research process cannot be purely objective and unbiased due to the researcher's role in the design, data collection, and interpretation, which influences the research process (Clark, 1998; Milch and Laumann, 2018).

## Results and Discussion

In this section, the resulting themes of the literature analysis are presented in a thematic map in three stages. The first stage represents the pre-change or background state, meaning what the organization is like before the introduction of the new technology. The second stage is the change process stage, which includes organizational interventions to support the ongoing change and human-technology interaction. The third stage represents the post-change or outcome of the technology adoption. A detailed overview of the stages, themes and subthemes, and references is provided in Supplementary Table 1.

The overview also shows the percentage of different sectors represented in the thematic map. The sector of management, business, and economics was represented the most, with 35 percent of all the references. In terms of region, North America (37%) was the prominent sector. As for the method, the references were mostly qualitative (45%). An overview of the descriptive analysis of the publications used for thematic map is provided in Supplementary Table 2.

### Pre-change Stage

In this section, the themes and subthemes in the pre-change stage are discussed. This is the state of organizations prior to technology adoption. The current state of the themes in the organizations have been shaped by past change processes and these themes play a role in future change processes. The main themes from the literature review that were placed under the pre-change state are organizational culture, organizational structure, leadership and management, and resources (see Table 1). Each of these themes consists of subthemes. A description of the main themes and subthemes is presented in Table 1.

**Table 1:** Themes and subthemes in thematic map for pre-change stage.

**Theme**	**Subtheme**
Organizational culture	Vision and goal
	Strategy
	Values and norms
Organizational structure	Power structure and work routineness
	Policies and regulations
	Cognizance
Leadership and management	Leadership style
	Participative management
	Sensemaking management amongst multiple stakeholders
	Emerging influencers
	Technology selection and human factor analysis
	Resource management and reconfiguration
Resources	External resources
	Internal resources

#### Organizational Culture

Organizational culture is a very broad theme that has been discussed expansively in the literature. It is the foundation of the organization and is essential as it permeates all the functions of the organization, including technological changes. There are different types of organizational culture (see Shin and Shin, 2020), but none of the frameworks captures all aspects of organizational culture. Organizational culture has been mentioned as the main barrier to change (Belisari et al., 2020). The main subthemes for organizational culture derived from the literature that are found to influence technology adoption are presented here: they include *vision and goals, strategy*, and organizational *values and norms*.

The subtheme of *vision and goals* is important as it sets the direction and focus of the organization. Vision refers to the long-term clear and coherent view of the future state of the organization. Having a common long-term vision across the organization inspires acceptance of changes to achieve the desired state, but if there are competing visions, there will be division as to what should be done and how (Taylor et al., 2015), which could impair the change process. The vision and the goals should ideally be coherent because goals help achieve the vision by forming the basis of objectives, policies, and organizational guidelines (McKenna, 2006). We believe that when planning to adopt a new technology, it is important to consider whether the organization has successfully established a shared vision and set of goals across its echelons that foster the adoption of new technologies. Furthermore, we need to consider whether the purpose of that particular technology is aligned with the vision and goals. If not, adjustments need to be made to the choice of technology. When the vision and goals are clear, respective strategies to adopt technological changes should be put in place to reach the goals. In addition to that, goal congruence between management and employees is important in facilitating technological change and overcoming possible political barriers to technology acceptance and eventual performance (Obal and Morgan, 2018).

The subtheme of *strategy* reflects on how an organization positions itself with respect to internal and external technological changes. We sorted the different strategies described in the literature into inertia, reactive, proactive, and isomorphism. Inertia refers to an intentional non-response to the changing environment because there is no threat or opportunity in adopting the change (Wang et al., 2015). A reactive strategy to change refers to the lack of an effective response mechanism to external changes, causing instability and avoidance of confronting the future (Miles et al., 1978). An organizational culture characterized by an external focus benefits from higher awareness and adoption of advanced technologies (Shin and Shin, 2020). A proactive strategy is about developing greater efficiency in adopting change or exploring the external environment for new opportunities, also known as a prospective strategy (Miles et al., 1978). Isomorphism is when organizations take up new technological changes due to normative, mimetic, or coercive pressure exerted by their external environment (for more details, see Parris et al., 2016). We believe that a proactive strategy could be the best approach, followed by isomorphism, to cope effectively with rapid technological advancements in the global market, while inertia and reactive strategies could eventually make the organization obsolete and unable to compete in the market. To enact the right strategy, the right norms and values should be created. This is discussed further under the next subtheme.

The subtheme organizational *values and norms* is an important part of the literature, as the success of technology adoption is partly due to showing respect for cultural norms during the process (Ford et al., 2016). Norms and values are the taken-for-granted guiding principles that help people understand what is acceptable and what is unacceptable. These patterns will be passed on to new members to guide them in their thinking, perceiving, and feeling (2, 1990; Moorhead and Griffin, 2004). Two of the most important norms and values regarding technological change found in the literature review are presented here. The first is the norm of learning and innovativeness. This will enable members to develop new skills and competencies that help these continuously learning organizations to undergo constant renewal (Tamayo-Torres et al., 2016). The term “knowledge management” was mentioned in the literature as a recommended practice by which a member's knowledge can evolve into team and organizational knowledge (Han et al., 2020). The more knowledge is built and shared, the more readily the organization will adopt new technologies and the more likely it is to survive. The second norm or value is the value of knowledge-sharing, which facilitates knowledge transfer and collaboration amongst stakeholders by removing barriers such as cultural differences and language barriers (Tseng, 2017), enhancing collaboration to meet the challenges of adopting new technologies. To enforce a constructive strategy toward technology change, organizations must reconsider their existing norms and evaluate whether these norms make employees motivated to try new approaches and solutions, share their knowledge, and learn from their co-workers. This could potentially create a bottom-up willingness to adopt new technologies rather than a top-down decision by the management, and increase technology acceptance and use.

Understanding organizational culture is important for many purposes. It is at the foundation of any change and, in this paper, we have tried to provide an overview and suggestions as to how vision and goals, strategy, and values and norms should be understood and aligned with the purpose of change. It is important to emphasize that having a vision for more advanced operation by means of technology, having organizational goals related to technology introduction and use, adopting a proactive strategy that encourages the employees to take more initiative in adjusting and using new technologies, and implementing norms of knowledge sharing and collaboration and innovativeness will facilitate ongoing and continuous technology adoption and use in organization.

#### Organizational Structure

When we have established the right the norms and values, the next step is to consider whether the organizational structure supports acting upon these norms. The structure of an organization determines how individuals work together toward a common goal. The most relevant subthemes to consider under this theme include *power structure and work routineness*, and *policies and regulations*.

The subtheme of *power structure and work routineness* refers to the hierarchical structure and diversity in work tasks. According to Bruns and Stalker (1961), organizations can be placed on a continuum ranging from a mechanistic to an organic structure. A mechanistic system is one in which tasks are highly specialized and communication is carried out through the chain of command. It is suitable in times of relative stability. An organic structure, however, is more suitable in times of change and a turbulent market (McKenna, 2006). An organic system is less hierarchical. Communication is not hierarchical. There is more task diversity and autonomy. Therefore, people are more engaged and innovative (Avadikyan et al., 2016) and there is better communication and coordination of tasks and staff at a lower cost (Bruns and Stalker, 1961; Miller, 1986; Bowen et al., 1989; Bahrami, 1992; Youngdahl, 1996). Other terminologies used for this concept in the literature include centralized vs. decentralized (see Wang and Feeney, 2016) and formal vs. informal power structures (see Peir and Meliá, 2003). It is worth highlighting that the relationship between organizational size and technology adoption is not clear, since it is argued that large organizations have more resources for faster technology adoption while small organizations have less bureaucracy and structural complexity that could slow down the adoption of technology (Han et al., 2020). We believe that when organizations have a more organic structure during technology adoption, the employees are more engaged and communicative. They are more likely to be innovative and to feel more secure and willing to share and acquire knowledge from others. This can enhance acceptance and adoption speed. However, as mentioned earlier, an organic structure works best during the time of change.

The state of change also requires having regulations and policies that would help the transition which brings us to the subtheme of organizational *policies and regulations*. Organizational policies should be adjusted to the circumstances arising from new technology to ensure a smooth adoption process. Organizations must be able to monitor and solve any potential problems and concerns that may arise during the process. This can be achieved through assembling governing bodies and project teams to develop policies for times of change that will address safety, security, and protocols for handling complaints (Wells et al., 2015), helping the adopters to adjust and to be compensated for the time lost and not punished for possible errors and compromised productivity during the change process.

#### Leadership and Management

“Leadership is a process by which an executive can direct, guide and influence the behavior and work of others toward the accomplishment of specific goals in a given situation” (Iqbal et al., 2015, p. 2). The role of leadership and management in innovation is essential, as reflected in the subthemes found in the literature review, including managerial *cognizance, leadership style, participative management, sensemaking amongst multiple stakeholders, resource management and reconfiguration, technology selection and human factor analysis*, and *emerging influencers*.

The subtheme of *cognizance* refers to the managerial ability to direct attention toward technological and market changes and to provide the necessary conditions to embrace change and remain competitive (Tamayo-Torres et al., 2016). The increased technological changes and complexities of the modern economy call for managers' constant scrutiny and investigation of the market (Eze et al., 2019). This also implies that managers themselves should have the cognitive ability to adjust to these changes and have a sense of efficacy in meeting the demands of restructuring, re-engineering, and modifying operational processes (Wang and Feeney, 2016). We believe that cognizance is important because it implies awareness of what is happening and how it affects organizational changes. With this awareness, management can adopt a suitable leadership style to navigate the change.

The next subtheme is therefore *leadership style*, the two most prominent being transactional and transformational leadership. This topic has been a widely studied topic in the management literature. Transactional leadership is “characterized by contingent reward and management by exception” (Schepers et al., 2005, p. 498) and is suitable for stable conditions. Transformational leadership, introduced by Bass (1985), refers to a style that is inspirational and motivates employees, raises their awareness, creates a long-term vision, and adjusts the system to foster creativity and productivity. As such, it helps with technology acceptance (Howell and Avolio, 1993; Yahaya and Ebrahim, 2016; Tseng, 2017). Transformational leadership was mentioned as the most suitable style for the implementation of complex technological changes, as characterized by Industry 4.0, which requires consideration of adopters' needs (Sony and Naik, 2020). Transformational leadership has a positive relationship with technology acceptance (Farahnak et al., 2020). Transformational leadership is related to participative management, which shows how this style can be put into action.

The *participative management* subtheme concerns the extent of management support and commitment, availability, and openness to continuous dialogue, leading to the success of organizational change (Andersen, 2016). The management shows support by being committed to the vision (Martins et al., 2016) and providing both the material resources and the internal political resources (Zhang and Xiao, 2017). Being committed, creating milestones, and celebrating achievements throughout the process enhances change momentum (Maali et al., 2020). A risk factor in the adoption process is the rapid pace of technology implementation compared to the organization's capacity and potential to adapt (Vrhovec et al., 2015). Management's participation will lead to an awareness of the increased pressures on the stakeholders involved. Consequently, they will be able to act so as not to seem apathetic in the face of the increased workload (Vrhovec et al., 2015). By showing awareness and consideration, the management can influence the perception of the stakeholders involved. This brings us to the following subtheme.

The next subtheme under leadership is *sensemaking management amongst multiple stakeholders*. Handling multiple stakeholders requires a certain level of soft skills (De Carvalho and Junior, 2015). A manager should map the external and internal stakeholders in technology adoption, consider their competing vision and goals that will lead to mixed opinions about using the technology (Taylor et al., 2015), and adjust their discourse. The leaders should understand their audience's cost-benefit evaluation and address the concerns of the stakeholders accordingly. In doing so, managing the perception of fairness is important (Jiao and Zhao, 2014). The new system may segregate a group of users due to an incompatible level of knowledge and skill, and unequal participation and training opportunities. This marginalization may, over time, create prejudice, and bias in the organization (Andersen, 2016) that influences the stakeholders' sensemaking. Therefore, management must address the concerns of stakeholders based on the different stakeholders' perceptions of costs, threats, and fairness through tactful communication (Costa et al., 2014) and the involvement of stakeholders in decision-making to avoid multiple interpretaions (Heath and Porter, 2019). The management may also appoint appropriate change influencers (Taylor et al., 2015) to better manage the sensemaking of multiple stakeholders.

The next subtheme is that of formal and informal *emerging influencers* (change agents, champions, opinion leaders). A change agent, or influencer, is a person charged with managing the change efforts for a group in which they have a certain level of influence, especially among those employees that are dissatisfied with the change process (McKenna, 2006). They are also referred to as opinion leaders and have been found to affect the technology adoption behavior of employees (Hao and Padman, 2018). These influencers can enhance or impede the diffusion and success of the change. They can inspire people through a tailored discourse (Kim, 2015), spread awareness, and promote the adoption of technological changes (Taylor et al., 2015). However, they may also emerge because of abdicated leadership and may steer the change process in their own desired direction (Andersen, 2016). This highlights the importance of managers' awareness of informal power structures in an organization and the management's close involvement in order to prevent derailment of the change process.

The next subtheme is *technology selection and human factor analysis*. Managing sensemaking is not just about the people, but also about selecting a technology that “makes sense” and is usable from the start. One of the more recent issues found to influence successful technology use in Industry 4.0 was concern about choosing a system that offers well-integrated cyber security measures. Sony and Naik (2020, p. 809) mentioned that “The successful implementation of Industry 4.0 will depend on the successful implementation of [a] cyber-security strategy.” This subtheme is about choosing the most suitable technology for a desired outcome and conducting the relevant human factor analyses. In the literature, a common problem was found to be that human factors received limited attention compared with technological aspects (Molino et al., 2020). Lack of consideration for human aspects jeopardizes successful technology implementation (Heath and Porter, 2019). “Technological development could benefit from including human factors experts from the project's outset to bridge the gap between the lack of relevant information and sufficient information on which to base development decisions” (Sætren et al., 2016, p. 595). This is crucial in the pre-change stage because insufficient evaluation of the new technology's usability and viability may lead to a costly failure. Consideration of human factors was found to ensure a more user-friendly system (Keyworth et al., 2018), better integration of the new technology into the existing workflow, and work process optimization (Heath and Porter, 2019). It was mentioned in the literature as a critical success factor in technology change implementation (Efremovski et al., 2018; El Hamdi and Abouabdellah, 2018). Viability analysis helps managers make better decisions by detecting opportunities and threats of the new technologies and adjusting the processes and services accordingly. One solution offered was for management to conduct pilot testing of the new technology to uncover potential future barriers before its full implementation (Keyworth et al., 2018).

The selection of suitable technology and conducting sufficient analysis depends on the available resources. Lack of resources can limit the choice of technologies and the ability conduct human factor analysis. This brings us to the next subtheme, which concerns the managers' role in allocating and reconfiguring assets and resources (Teece, 2007). As technology selection and analysis, along with reconfiguring and allocating adequate resources, are parts of managerial responsibilities, these subthemes are presented together here. However, the nature and range of resources will be discussed as a standalone theme due to its significance and breadth. There is a certain overlap between the themes of leadership and management and resources, and a clear-cut distinction may undermine the fact that the themes and subthemes identified at each stage exist and occur simultaneously and take shape parallel to one another.

Another important subtheme of leadership is *resource management and reconfiguration*. An organization has various types of both internal and external resources at its disposal. These must be combined and reconfigured in order to reap the most benefit from the technology adoption process (Pace, 2016). “Reconfiguring and managing resources are indicators of the firm's ability to create a better ‘fit’ with its environment” (Pace, 2016, p. 411) and develop competitive capabilities through strengthening and adjusting resources (Teece, 2007). This is referred to as sensing and seizing, which reflects the manager knowing where to invest to create value from the innovation (Pace, 2016). Augier and Teece (2008, p. 1190) defined dynamic capabilities as the ability to “shape, reshape, configure, and reconfigure assets” to be profitable in the face of altering markets and technologies. Management must first identify all the available resources that could help with the technology adoption process. However, there is not enough discussion in the literature of how resources should be reconfigured during technology change as opposed to other changes.

#### Resources

The next main theme found in the literature is resources. Based on the analysis, we consider resources to include material resources (technical resources, infrastructure, budget) and immaterial resources (employees' knowledge and skills, organization's political resources and partnerships, time). It has been found that an organizational capability to be agile in responding to changes is influenced by tangible and intrinsic resources (such as accumulated experience), internal resources, and external facilitating resources (Ali et al., 2018). This is discussed further under the subthemes of *external resources* and *internal resources*.

The subtheme of *external resources* is about the resources outside the organization that can help in adopting a new technology. They are crucial in allowing an organization to take advantage of its external environment (Pace, 2016). External material resources may be in the form of sponsorships or funding bodies, or even being able to use others' premises to carry out certain tasks or to outsource some operations. External immaterial resources are mainly the network of knowledge sharing, which in the long term will reduce the external knowledge absorption costs (Antonelli and Scellato, 2013). This network can comprise professional network groups, consultants, or technical support team that can help with technology implementation and use.

The next subtheme under resources is *internal resources*. Internal material resources include the available technological infrastructure and budget. Resources in the form of budget can be allocated to enhance technology adoption through incentive systems that reward and motivate employees' effort to embrace new technologies (Vaishnavi et al., 2019). Having an adequate infrastructure is important in technology adoption because the extent and the quality of the infrastructure determines the attitude and behavior of the adopters (Langstrand, 2016). This implies that having the necessary conditions to introduce a new technology or to incorporate a new technology into an existing system makes it easier for users to accept it because there are fewer problems to be solved. The adopters are more ready and more likely to accept the technology when they know that the organization has the infrastructure for the new changes. The previous technology becomes the foundation upon which new technologies are built, and technological infrastructure accumulates over time (Gillani et al., 2020).

Internal immaterial resources mainly comprise the organization's human resources, meaning the employees, and their attitude and aptitude, prior to the adoption of new technological change. Attitude and aptitude can either slow down the process or speed it up based on how they are shaped and utilized. The employees can hasten and enhance the process if they are ready for technology changes. Zhang and Xiao (2017, p. 433) define citizen readiness as “a state of mind—a citizen's predisposition and likelihood to try new technology services”; the authors posit that technology cannot be absorbed if people are not ready. The same can be applied to employees in an organization.

Aptitude refers to the potential and capabilities that an organization possesses through its employees' tacit and explicit knowledge, their awareness, skill level, and expertise (Naor et al., 2015; Vrhovec et al., 2015; see Freeze and Schmidt, 2015). Attitude is a collection of many constituent elements shaping how employees view the organization. The most relevant elements shaping a person's attitude include personality traits, where openness to experience and extraversion have been argued to enhance change acceptance (Huang, 2015). Another element is perceived psychological safety, which is having a sense of security in the face of new developments. It is the result of the employees' perceived cost and benefit, perceived personal valence (Armenakis and Harris, 2009), perceived threat (Bala and Venkatesh, 2016), and perceived loss of control due to new changes. Perceived procedural and outcome fairness, trust in the management's intention behind the change (Nielsen and Mengiste, 2014), and having a sense of efficacy to cope with new changes (Sætren and Laumann, 2014) can influence attitude toward a change. Furthermore, subjective norms, or “the perceived social pressure to perform or not to perform the behavior” (Ajzen, 1991, p. 188), influence the intention to adopt a new technology (Bayerl et al., 2013). Employees' commitment to the organization's norms motivates them to adopt the change (Vella et al., 2013).

When introducing a new technology, the level of aptitude of the employees must be considered to see if they have the necessary knowledge and skills to adopt the change. If they do, they will be more ready to accept the new technology; if not, organizations must consider interventions such as training that improves the aptitude of employees. It is more difficult to evaluate attitude of employees than to evaluate the aptitude of the employees. Organizations cannot change the personality traits of employees, but when introducing new technologies, they can divide and appoint tasks that better suit their personalities. For example, those with higher levels of openness and extraversion could be suitable change agents. Perceived psychological safety, fairness, and efficacy should be investigated prior to and during the technology change. This can be done through anonymous opinion surveys or forums, as well as in-person information sessions. Based on the results, interventions can be planned to address the concerns raised by employees.

### Change Process Stage

In this section, the change process stage and the themes found in the literature that were placed under this stage are presented. The main themes are organizational interventions and human-technology interaction. Interventions include communication, participation, and training in order to smoothen the ongoing change process and enhance the quality of technology adoption. At the same time, a human-technology interaction process is taking place. This is when people are engaged with the technology and are trying to make sense of it based on their attitude and aptitude, the technical features of the technology, and their perceived level of organizational support. These main themes and their subthemes are presented next. An overview is provided in Table 2.

**Table 2:** Themes and subthemes in thematic map for change stage.

**Theme**	**Subtheme**
Organizational intervention	Communication about the technology change
	Participation of key actors and users in technology adoption
	Training for key adopters and mediators
Human-technology interaction	Technology sensemaking
	Physical proximity and accessibility
	Technical support

#### Organizational Intervention

This theme is an important theme in the literature. It refers to the initiatives that organizations take to reinforce the change process. These measures help respond to the challenges of technology adoption and improve the process. Interventions are tailored to the specific context and time, and to the specific technology and its features. It is the presence or absence of these interventions and the extent of the quality and timeliness with which they are offered that make them enablers or barriers. These interventions are discussed under the subthemes *of communication about the technological change, participation of key actors and users in technology adoption*, and *training for key adopters and mediators*.

The subtheme of *communication about the technological change* represents an essential intervention. Organizations should be very strategic about how they convey information about technological changes. Their discourse must be aligned with the organizational culture, tailored to the audience, consistent, continuous, and honest. This will enhance employees' sense of trust and perceived fairness, and reduce feelings of anxiety about the technology change. Timing is essential. Communication should take place in the initial phases of the introduction of the technology, or even as early as the selection decision. Cross-communication early on is important “in order to set a common goal for harmonization, coherence and collaboration” (Kierkegaard, 2015, p. 152) between stakeholders. This leads to early role clarification for all the stakeholders, and helps to avoid confusion and uncertainty. The choice of communication channels and networks is also important. There are multiple official and unofficial networks within organizations. The different stakeholders may also communicate with their respective external partners. Managing communication through these channels and networks is essential in technology sensemaking across the organization's ecosystem. Communication will result in increased awareness, knowledge, and collective problem-solving. Another important aspect of communication is homogeneity across various receivers in terms of frequency and thoroughness, which is important in perceived fairness and trust.

The next subtheme is named *participation of key actors and users in technology adoption*. Communicating about a technology provides a platform for all the key actors to voice their opinions, to be acknowledged, and to contribute to the betterment of the process (Jensen and Kushniruk, 2014), and participation logically follows. Participation is about empowering end users and key actors, promoting their engagement and commitment (Rizzuto et al., 2014). In the long term, this will mean more problem solving at the beginning and a better, faster, and more cost-effective adoption process. There is also a higher likelihood of continuance of use and increased psychological safety (Antonioni, 1994 as cited in Johannsdottir et al., 2015).

The next subtheme is *training for key adopters and mediators*. Training is an important contributor to successful change implementation (Petit dit Dariel et al., 2013). This intervention is particularly effective in overcoming knowledge and skills gaps, and helps to enhance self-efficacy, reduce technostress (Molino et al., 2020), and improve technology adoption. By reducing the perceived complexity, it can enhance PEOU and technological efficacy, leading to more positive attitudes as it enhances sensemaking (Takian et al., 2014) and contributes to the success of technology adoption (Herbert and Connors, 2016).

To be effective, training should be carefully designed and tailored to the receivers. The choice of discourse and the phrasing of the objectives of the training are very important to create a realistic expectation of what the training can lead to. The trainers need to be carefully chosen as they are the first mentors in guiding employees in the use of the technology. It is important to deliver knowledge in such a way that people can utilize it to better accomplish their task objectives (Mühlburger et al., 2017). It is also mentioned in the literature that adequate adjustment time is needed to allow the users to familiarize themselves with the technology in the initial stages of change (Keyworth et al., 2018). Furthermore, the stages in training and the actors are important considerations. For example, it was mentioned in the literature that it is best to allow employees to familiarize themselves with the technology for less complicated and risky tasks and then move on to riskier contexts (Thomas and Yao, 2020). Training is a strategic investment that strengthens an organization's internal resources by targeting employees' aptitude and attitude toward current and future technological changes. Training should not only focus on technical skills, but also on soft skills as “they represent protective factors in changing situations” (Molino et al., 2020, p. 10), especially with regard to the new industrial and technological development era of Industry 4.0. Enhanced positive attitude, along with the necessary skills, leading to technology acceptance and use can, in turn, create a feeling of greater work engagement and motivation among employees, and so improve performance. The knowledge and skills acquired will become part of the stored knowledge and infrastructure of the firm. Training is considered to be “an important incentive for work, even more important than extrinsic rewards or compensation” (Shirish et al., 2016, p. 1120), and when it comes to incentives, “training and development is the most prized benefit” (Han and Su, 2011, p. 3). However, one issue mentioned in the literature is the importance of standardization of training in a sector in order to avoid training employees “in various ways, using different means, and achieving different levels of proficiency” (Kumar et al., 2018, p. 36).

Training is an important tool to influence organizational culture when it comes to resistance to change. Training should have a clear learning objective, and it should also clarify the mechanism by which it will enhance task performance and reduce the perception of risk and threat (Escobar-Rodríguez and Bartual-Sopena, 2015). The result will be smoother technology adoption and assimilation.

#### Human-Technology Interaction

Technology does not operate in a vacuum and it cannot be isolated from its social context. The use of technology is very much dependent on the user experience. Therefore, this theme is about how this user experience is formed through interaction with the technology, but also as a response to how the organization manages technology changes. Human-technology interaction will determine the fate of the technology and its further use. The most relevant underlying mechanism of this interaction is reflected in the subthemes presented in this section. These subthemes include *technology sensemaking, physical proximity and accessibility*, and *technical support*.

The subtheme of *technology sensemaking* encompasses the broad emotional and cognitive processes involved in the evaluation of the new technology adoption process. Employees evaluate how the technology has been put to use—volition vs. coercion in use and the perceived fairness during the process (Jiao and Zhao, 2014). The employees evaluate those who developed and introduced the technology and the management based on what the employees' attitude is toward the developers and managers. Employees also evaluate whether there was an urgent and legitimate need for this technology and whether the technology itself was a suitable choice. They evaluate the usability of the technology (is it easy and fast to learn or is it complex and time consuming?) and the attributes that make the technology more attractive to use. These attributes are summarized by Rogers (2003, p. 223) as “(1) relative advantage, (2) compatibility, (3) complexity, (4) trialability and (5) observability.”

The attribute of relative advantage presented by Rogers (2003) is about the superiority of the innovation or technology compared to the previous version. This attribute is closely linked to other concepts presented in the literature, such as PU and PEOU, which influence the intention to use the technology (for more detail, see Davis et al., 1989; Vella et al., 2013; Escobar-Rodríguez and Bartual-Sopena, 2015; Goldkind et al., 2016). PEOU is also related to technological efficacy, which is about having a sense of capability based on one's technical skills and the procedural knowledge that is needed, and being confident that new skills and knowledge can be acquired (for more details, see Hopp and Gangadharbatla, 2016; Martins et al., 2016). A lack of technological efficacy could lead to the experience of technostress and possibly job dissatisfaction and resistance to change (Freeze and Schmidt, 2015). PEOU may be influenced by how sophisticated the technology is (Ghorab, 1997): for example, if the technology presented is an early prototype, it will not yet be well-developed and will not be easy and smooth to use. This can lead to a lack of usability (Bourrie et al., 2015), leading to a negative evaluation of the technology.

The attributes of compatibility and complexity (Rogers, 2003) are also referred to as assimilative-disruptive and incremental-radical technology in the literature. Complexity is about the degree to which the novel features require learning in order to use the new technology. Compatibility is about being able to integrate the technology into current tasks without disturbing the current methods due to similar technical standards (Nagy et al., 2016). It has been found to be an important factor in technology adoption (Hanafizadeh et al., 2014). The more complex the technology, the more cognitive and emotional resources are needed to learn how to work with it and to cope with the challenges. Employees may evaluate whether it is worth it to meet the demands of the complex technologies. In doing so, they also evaluate the task-technology congruence. This is about whether the technology actually does what it is supposed to do and the extent to which it fits with the deliverables defined for the task with improved efficiency (Vest and Kash, 2016) and effectiveness (Singh, 2017). If it is worth it, employees will be willing to meet the demands of adopting new technology. Literature has shown that users evaluate if the technology helps them achieve their goal at the desired level of quality and without increasing the time needed to perform the task (Barrett, 2018).

The remaining attributes mentioned by Rogers (2003) include trialability, which is about being able to experiment with the technology, modify, and reinvent it as it evolves. The attribute of observability refers to being able to see the results of the use of technology and become motivated to use it.

Another subtheme that influences the human-technology interaction is *physical proximity and accessibility*, which is about easy access to the technology and proximity to developers and managers. Proximity is important because it enhances continuous communication and dialogue between key actors, leading to work process improvement that will, in turn, enhance technology acceptance and use. Lack of accessibility decreases PEOU (Vella et al., 2013), and unequal proximity creates organizational bias over time due to inconsistent communication, mutual irritations, and compromised technical support.

The availability of *technical support* facilitates adoption. It helps with managing technostress, promotes learning and knowledge sharing, and enhances psychological safety. It shows commitment on the part of the management, and the social support that will help overcome all kinds of challenge during the adoption process (Freeze and Schmidt, 2015; Wells et al., 2015; Avadikyan et al., 2016).

We believe that the change process stage is crucial in technology adoption and use. Interventions can shape the attitude and improve the capabilities of users regarding the technology and the organization. They can set the tone for constructive communication and participation, and, over time, create the cultural norms and values that enhance technology acceptance and innovation. This is the time either to make up for the organization's history of past technology implementation failures or to build on a history and culture of successful technology implementations. Past experiences of failure pose a risk to new changes and create uncertainties (Han et al., 2020), while past successes facilitate new technology changes (Kumar et al., 2018). We believe that by knowing the pre-change stage very well and carefully designing the interventions, organizations can influence the human-technology interaction and lead the change process toward a desired outcome. This is the time to compensate for the shortcomings of the past and to create a foundation for current and future technology adoptions.

### Post-change Stage and Outcome

The next stage of technology adoption is the post-change stage, or the outcome of the technology adoption process. Every organization is different, and the dynamics that they have gone through in the previous stages result in a range of responses in this stage, from resistance to acceptance and from use as intended to non-use. Resistance and acceptance are not mutually exclusive categories. Rather, we believe that the degree of resistance and acceptance can be placed on the spectrum of technology adoption outcomes. Behavioral responses on a unidimensional spectrum can range “from adoption to aggressive resistance (adoption, neutrality, apathy, passive resistance, active resistance and aggressive resistance)” (Klaus et al., 2015, p. 58). It may also be that only some aspects of the technology are accepted, or that some groups in the organization adopt it and some do not. Therefore, there is a range of possible outcomes. The major subthemes found in the literature that reflect on the prevalent responses are presented in the following section. Table 3 offers an overview of the themes.

**Table 3:** Themes and subthemes in thematic map for post-change stage.

**Theme**	**Subtheme**
Resistance	Aggressive non-use
	Workaround
	Involuntary non-use
Acceptance	Assimilation and use as intended
	Customization and technological evolution

#### Resistance

Bovey and Hede (2001) describe resistance as a defense mechanism due to an emotional reaction to change. Barrett (2018, p. 61) stated that “resistance can manifest behaviorally—protesting or complaining about the change, cognitively—believing the change is harmful or negative, and/or affectively—expressing negative emotions about the change such as fear or anger.” There may be resistance to new solutions because the existing solutions have already proved to be successful. This way of thinking is referred to as the competency trap in the literature (Cichosz et al., 2020). Resistance ranges from passive to active and from covert to overt, and it is influenced by previous experiences of learning and using similar technologies (Freeze and Schmidt, 2015). It can result from low employee motivation and increased ambiguity resulting from changes in work performance measures and conflicts with employees' innate value system (Barrett, 2018), causing goal conflict (Obal and Morgan, 2018). It can also be classified as institutional conservatism or individual (Cichosz et al., 2020). Resistance can range from intentional to unintentional forms of resistance (Andersen, 2016). The different resistant behaviors are presented in the subthemes of *aggressive non-use, workaround*, and *involuntary non-use*.

The subtheme of *aggressive non-use* refers to when employees openly protest and resist using the new system. As Lapointe and Rivard (2005, p. 467) state, “aggressive resistance behaviors such as infighting, making threats, strikes, boycotts or sabotage seek to be disruptive and may even be destructive.”

Another form of resistance is *workaround*. This is an indirect form of resistance displayed by working outside and around the system. It is often unconscious and is due to a lack of knowledge of the system, having anxiety about using the new system, lack of infrastructure to support the use of the new technology, material constraints, and feeling that the use of the technology slows down the work and does not provide any value in performing the primary tasks (Ferneley and Sobreperez, 2006). The conditions that can lead to workarounds include enforced proceduralization and non-engagement with the system (Ferneley and Sobreperez, 2006; Freeze and Schmidt, 2015; Vrhovec et al., 2015). Workarounds are not a direct form of resistance because they are more likely to “emerge from users completing the assigned tasks to the highest quality, within the imposed time frame and in a professional manner all within their day-to-day activities, especially during stressful days” (Choudrie and Zamani, 2016, p. 144). However, Heath and Porter (2019) reported that workaround behavior displayed by adopters was either informal and on the individual level, or formal and on an institutional level in response to government-enforced change. Furthermore, workaround is not necessarily a deviant behavior, as posited by Malaurent and Karanasios (2020), but rather a learning process that intends to find a way to accomplish a goal and complete a task. This kind of deviations are therefore “harmless workarounds that can then be classified as essential or hindrance workarounds” (Choudrie and Zamani, 2016, p. 144) and the intention for this type of workaround is to get the primary tasks and goals done.

The next subtheme is *involuntary non-use*. This is when employees without sufficient access and proximity to managers and technical support experience increased difficulty in using the technology. Due to the lack of communication, they become isolated, marginalized, and unable to use the technology effectively despite their intention to do so (Andersen, 2016).

The notion of resistance through involuntary non-use implies that there can be resistance through involuntary use, which can be understood in line with what is referred to in the literature as forced adoption (Zhou, 2008). This is when an organization has adopted a new technology and the employees are pressured directly or indirectly to adopt and use the technology (Zhou, 2008). This could imply that the technology is not inherently accepted or liked, but there is also no way around using it. Although this notion was not directly derived from the data analyzed, we believe that it is worth considering.

#### Acceptance

Like resistance, the acceptance response can be placed on a spectrum according to how the technology is being used. The subthemes found in the literature under this theme include *assimilation and use as intended*, and *customization and technological evolution*.

The subtheme of *assimilation and use as intended* refers to a scenario where employees are convinced that the new technology is needed and it enhances their job performance. They trust those who have implemented it (Sætren and Laumann, 2014). They have been able to acquire the knowledge and competencies required to use the technology in the way it was intended in their routine tasks.

The subtheme of *customization and technological evolution* refers to a case where employees have not only assimilated a technology into their routine tasks, but they have even modified, customized, or tailored it to enhance performance and effectiveness. Therefore, they have taken it even further than was intended in the first place. This is characteristic of highly motivated and innovative work units (Wells et al., 2015; Ford et al., 2016). Customization can be positive or negative depending on the direction and the intention of the customization. Furthermore, the context in which customization takes place within an organizational ecosystem plays a role in the outcome. In certain contexts, customization in the form of creative use of technology could be beneficial, but in a safety-critical context, customization that is not planned and is not according to protocols could lead to hazardous outcomes. However, it should be mentioned that Malaurent and Karanasios (2020) contrasted customization with workaround, stating that workaround is the employees' innovation of using technology in an unexpected way while customization is induced by the organization in a top-down manner.

We believe that it is important to decide at the change process stage where on the spectrum of acceptance and resistance, use and non-use, we want to be based on the nature of the task and the industry. For some tasks, flexibility is appropriate and users can customize their use of the technology. If that is the case, allowing some flexibility in use and not punishing the users for their response could give them time for transition into the use of a technology in a way that fits their task and reduces resistance. For other tasks or industries, there is no safe alternative to the predetermined use of technology and no flexibility can be accepted. This needs to be clarified, and support and training needs to be given to help users so that we can reach the desired outcome. While use and non-use behaviors are easier to observe, resistance and acceptance need more effort from the organization to adjust users' attitude the through interventions, interaction, and adjustments to the culture and structure of the organization. Therefore, all the stages are interconnected, and all stages influence one another.

Bhattacherjee et al. (2017) provided a taxonomy of use response and acceptance of organizational IT changes when adoption is not under full volition. Their acceptance category includes engaged and compliant responses, while their resistance category includes reluctant and deviant responses. However, they also acknowledge that there is no dichotomy in acceptance and resistance behavior. They state that positive and negative feelings and responses can co-exist.

### External Environment

The external environment influences an organization continuously throughout technology adoption and use and is not limited to one stage in particular. The external environment can be best understood through the PESTEL framework. This refers to the political, economic, social, technological, and legal elements in the organization's environment (Bayerl et al., 2013). External influences are important triggers of organizational technology investment choices (Kapoor and Lee, 2013). They may be induced by general trends in the society that shape the preferences of citizens and service providers, and these social norms or preferences influence technology choices (Poba-Nzaou et al., 2014). Globalization and rapid technology advancements have been found to impose competitive pressure on organizations for survival. Governmental pressure has also been seen as a key factor in technology adoption (Aldossari and Mokhtar, 2020; Han et al., 2020). Legal bodies and other socio-political actors also influence technology trends (Nielsen and Mengiste, 2014). Market competition (Bhuyan et al., 2014) and customer demands push an organization to adopt a technology, even more so than the regulatory bodies and managerial pressure (West and Berman, 2001). In addition, the external network of organizations influences the adoption decision and success in a positive way through fostering trust, such that the more organizations that have adopted a technology externally, the more external support and standards will be available to the adopting organization (Han et al., 2020). We believe that the external environment and the organization are in constant interaction as part of the organizational ecosystem, and all the stages of change are influenced by the external environment. This theme provides the context in which technology changes take place.

The thematic map provides an overview of the relevant themes and subthemes in technology adoption process, in stages and it enables us to see when the themes are antecedents to next themes and when they are consequences or aftereffects to previous themes. The timely consideration of the themes in accordance with the internal and external context of the organization, turns the themes into enablers and otherwise they will become barriers to technology adoption. The outcome of each stage become input to next stage and this process continues in a circular manner for ongoing technology adoptions.

## General Discussion

The aim of this paper is to analyze the existing literature (1) to determine the main themes that were found to be influential in organizational technology adoption; (2) to organize the themes and subthemes in stages so that it is clear when themes are antecedents and when they are after-effects; and (3) to present and discuss how the themes can be enablers or barriers. The thematic map demonstrates a system where all the elements are in a dynamic interaction with one another and are not mutually exclusive nor independent. Rather, they can influence one another at any time, and changes in one component influence the other components through the lifecycle of the technology adoption in the organization. At the pre-change stage, this includes the organizational culture, organizational structure, and management practices. The acquisition, allocation, and reconfiguration of resources is essential in successful technology adoption. Internal human resources—including the skills and knowledge of the employees and their attitude that is shaped at the pre-change stage but also during the change stage as the result of interventions and interaction with the technology—influences how they will use or misuse the technology, or the outcome at the post-change stage. The response will be oriented on a spectrum ranging from acceptance to resistance. The post-change state will be fed back into the organizational ecosystem and will affect the preconditions for the next changes that occur in the organization. The presence or absence and the extent of the availability, quality, and timeliness of each theme can transform that theme into an enabler or a barrier. A theme or subtheme that is considered and taken into effect at the right time, in the right manner, and in line with the context becomes an enabler. Otherwise, when it is absent, when it is not given enough attention and is not offered at the right time with the right quality and in alignment with the context, it becomes a barrier. The themes are antecedents of the next stage of the thematic map and are after-effects of the previous stages or the previous adoption processes. A positive outcome and successful technology adoption will enhance future technology adoptions, while a negative outcome and an unsuccessful adoption will impede it, as if is stored in the organizational history and the collective attitude and aptitude of the employees. It undermines their trust in the management. The dynamics of this are grounds for future research.

The theoretical implication of the thematic map is the consolidation of the existing literature into one comprehensive overview while organizing the findings into stages of the technology adoption process. This clarified the antecedents and after-effects at each stage of change, which were ambiguous in the existing literature. The importance of timing was therefore implied. Furthermore, we elaborated on how issues at each stage can act as enablers or barriers depending on how well they are managed. While many theories and past studies have treated the technology adoption and management process as a singular, linear, and static process, we emphasized that it is a continuous, circular, and dynamic process. The suggestion that stages and themes influence one another is in line with the study by Eze et al. (2019), who suggested that the different factors or issues at each stage are not static, meaning that these factors or issues can induce different dynamics at different stages. We also emphasized the importance of continuous consideration of the external environment surrounding the organization and the broader sociotechnical system.

Since this map is based on the existing literature, the findings are in line with the literature. However, it can be argued that technology adoption models—especially those on the individual level such as TRA, TPB, TAM, and UTAUT, that emphasize technological features and individual attitude—are in line with the change stage subtheme of human-technology interaction and organizational interventions such as communication and training that can influence attitude. Firm level models such as DoI and TOE expand to cover the role of leadership and management, therefore overlapping with the pre-change subtheme of leadership and management, the subtheme of organizational culture and structure, and, for TOE, the external environment that was omnipresent in the thematic map. However, none of these models fully covered all three stages and subthemes, and we do not have a clear indication of how the post-change outcome will be significant for future technology adoptions. Despite the commonalities, these models are still not able to capture all the complexities of Industry 4.0 technology adoption processes. This is because every industry is characterized by its own distinct contextual features that can affect the change process in different ways (Aremu et al., 2019). Nevertheless, the fourth industrial and technological revolution will eventually lead to a demand for a more global standardization of processes and technologies. Therefore, the theme of the external environment should expand to account for these changing trends. None of these aforementioned models clearly indicates the importance of organizational flexibility and resilience in adjusting to the dynamic and complex changes characterized by Industry 4.0. The thematic map highlights the dynamic nature of the process, but it should also expand its scope to identify and incorporate resilience, flexibility, and agility into the organizational culture and organizational intervention subthemes.

The change management models are more in line with the pre-change stage subthemes of leadership and management, and resources, and the change stage subtheme of organizational intervention. Perhaps the closest model to the stagewise thematic map is the Lewin/Schein model of change. It also includes three steps or stages of change (unfreezing, change, refreezing), consistent with the pre-change, change, and post-change stages of the thematic map. However, the Lewin/Schein model is more focused on the psychological mechanisms involved, such as dealing with learning anxiety or redefining a self-concept. The mechanisms or processes involved in each stage are not in exactly the same order as in the thematic map. For example, training in the Lewin/Schein model is emphasized more in the unfreezing stage, while in the thematic map, it is an organizational intervention during the change stage. Nevertheless, we do acknowledge that there is no one correct way of implementing technological change. Furthermore, the refreezing stage involves habituation and maintaining the new behavior achieved through the change that was implemented. However, the thematic map does not suggest a refreezing, but rather a dynamic system where the outcome of the change will serve as the input into new and ongoing technological changes in the organization and will impact the momentum of the change.

The study by El Hamdi and Abouabdellah (2018) also indicated a stagewise division and a focus on three areas during change: people, process, and technology. Their proposed structure was based on enterprise resource planning projects and may not cover all the complexities involved in Industry 4.0. Similarly, most of the studies are from the IS field (Eze et al., 2019) and have focused on the individual, the firm, or the technology, while the combination and the “fit” between the variables is less explored (Aremu et al., 2019). This is quite challenging, as creating a fit between components of a dynamic stagewise model can be very complex in broader sociotechnical systems (Oliveira and Martins, 2011; Culot et al., 2020). An organization as a system is too complex to be simplified into quantitative and predictive relationships between a limited number of variables. Organizations are complex systems, meaning that they are open, constantly interacting with their environment (Le Coze, 2005) and mutually influencing one another, and evolving over time. They are dynamic and adaptive, meaning that it is difficult to predict the effects of technological changes because of the myriad elements involved and their interactions within the whole organizational ecosystem. Therefore, as Le Coze (2005, p. 623) states, “organizations are difficult to predict through quantitative models: the interactions and the number of autonomous variables are too high to be put in equations.” In order to explore which themes within this complex system can play a role in the technology adoption process, therefore, a qualitative approach was deemed appropriate for mapping the themes at each stage.

With regard to the practical implications, we do not attempt to prescribe one right way, but rather recommend that before initiating change, the stages should be carefully considered to get the desired outcome. This thematic map is a tool to reflect on the current state of the organization and see where the organization stands with respect to an ongoing change. Based on this information, we can intervene to compensate for shortcomings that are found and/or strengthen processes where this is needed. This map also helps to reflect on the desired outcome and the acceptable level of flexibility in use. Finally, this map is a reminder that the outcome becomes the input for the next technology adoption, meaning that the after-effect becomes an antecedent of future changes in the organization.

### Limitations

Most of the reviewed literature in the thematic map stems from the fields of management, health, and information system and computer sciences, and it mostly represents the North American and European regions. Other sectors and areas of research may be underrepresented because they either do not focus on this topic or have too narrow a focus and a specific research scope that could not be incorporated in the thematic map. Other regions that may differ in culture, structure, and technology infrastructure are not equally represented. Technology adoption and usage may be defined and evaluated differently in other regions.

In conducting this research, we acknowledge the limitations of including and presenting all the research done on the themes in the literature. We acknowledge the role of the researcher in reflecting on and analyzing the data and that absolute objectivity cannot be claimed. We attempted to present the data analysis and results in a detailed manner so as to allow for transferability of the data to many organizational contexts. We do acknowledge that choosing the English language may have consequences on how biased or representative the research is or the extent to which it is communicated as intended.

We acknowledge the consequences of determining a degree of saturation that we considered enough as no new codes or themes were emerging. It is not possible to dismiss the potential for having come across new codes and themes had the data collection and analysis continued. Furthermore, even though new codes and themes were not being derived from the articles that were included in the analysis, it is possible that more articles would have contributed to the depth and diversity in each code or theme. Furthermore, most articles were representative of the management and health sectors. This means that saturation may have been reached for these sectors while other sectors may have been less well-represented than they could have been. Therefore, there is a limitation in establishing an equal degree of saturation per sector. However, since the aim of this paper was to create a thematic map that serves as an overview, providing an in-depth and broad account of each theme would have been beyond the scope of the paper. Furthermore, we acknowledge that removing certain articles prior to data analysis could have diminished the breadth and depth of the codes and themes in the model. Therefore, exclusion criteria for such studies can always introduce a level of bias in the articles selected. The process of selecting and coding the data was carried out individually, but the development of search terms and the development of the thematic map and the iterative categorization of the subthemes and themes was carried out in continuous discussion with the research team. The individual selection of publications could potentially create a bias in the evaluation based on primary screening stages. However, the iterative process of discussing and categorizing the themes and subthemes with the other researchers in the group enhances confirmability through peer debriefing.

## Conclusion and Future Research

This paper has provided a thematic map of the existing literature consisting of different theoretical frameworks, findings, and suggested interventions in one stagewise overview. The importance of timing is emphasized through the division into stages. The distribution of relevant themes and subthemes at each stage makes it easier to conduct problem-sensing and -solving, and to create subprojects that can be delegated in each stage for better change management. The issues at each stage are antecedents to the next stage. The issues in the following stages are after-effects or consequences. Whether the antecedent enhances change (an enabler) or impedes change (a barrier), depends on how those issues are considered, evaluated, and managed in the context. If they are managed well so that antecedents act as enablers of change, the outcome of the post-change stage at the end of the first cycle will be fed back into the next technology adoption round and it will increase the momentum of future technology adoptions. Otherwise, the mentioned issues will decrease the momentum. The stages are dynamic and influence one another. Therefore, the process of technology adoption requires continuous evaluation and adjustment. The goal should shift from a desired outcome at the post-change stage to enhanced momentum in technology adoption and increased adaptability to a changing environment.

The potential future research agenda could target a number of trajectories:

1. Future research can be a systematic literature review, including more databases and with adherence to the required protocols for a systematic review (Bramer et al., 2017).
2. Future research can also include meta-analysis, along with quantitative methods to investigate whether there is a relationship between themes proposed in the thematic map. Each stage can further be zoomed in and investigated in greater depth as separate topics. This is helpful to understand better the potential relationship and the nature of the relationship between the different themes and subthemes.
3. In the future, we could test the proposed framework for technology adoption stages and themes in order to qualitatively evaluate the organization during technology changes. This would allow us to build on the framework, refine the themes, and incorporate new themes and subthemes. This is possible by focusing on possible subprojects at each stage and creating guidelines for each stage of the technology adoption, identifying the key actors, and evaluating the best practices that would fit the internal and external context. A continuous evaluation and reassessment of the model in this way, will also enable us to track and understand the changing trends in different technological eras, such as the specific challenges and practices of Industry 4.0.
4. Future research should also focus on the standardization of technology and work processes. Global standards may exert a pressure on the organizations to change their culture, structure, and practices in order to adjust. These challenges deserve further attention.
5. Future research should also investigate whether it is possible and useful to have standardized methods of evaluating organizational technology change. There are a variety of tools, indexes, measurement methods, and typologies for each theme in the thematic map. These measures have been used for evaluation and assessment, but they are not consolidated and standardized into one evaluation checklist. There has not been one single exhaustive method of measuring and advising what organizations can do because every context is very different. Future research could look into figuring out if there can be one checklist that can be customized to different contexts, provide an overview of the organization's current state and help the organization become more flexible, resilient, and adaptive to their complex and dynamic internal and external environment.
6. Future research should also consider the effect of further digitalization and automation, the translation of data into information, and the instant availability of information to users, on the individual work motivation and performance, the organizational structure and culture, and the society. Therefore, future research should not only consider the top-down influence of the environment on technology, but also the bottom-up influence of technology on the organization and society and recognize that the technology adoption process is no longer linear.

## Author Contributions

MS conducted the data collection, analysis, and writing and revisions. KL and MS were involved in discussions on the research topic and question and the strategy for the literature search and participated in intensive discussions about the results and the overall manuscript. MRS was involved in planning and revisions. All authors contributed to the article and approved the submitted version.

## Conflict of Interest

The authors declare that the research was conducted in the absence of any commercial or financial relationships that could be construed as a potential conflict of interest.
