# Supplementary material for: Stagewise Overview of Issues Influencing Organizational Technology Adoption and Use
Source: Front Psychol. 2021 Mar 17;12:630145. doi: 10.3389/fpsyg.2021.630145 (PMC8009967; doi:10.3389/fpsyg.2021.630145)
Supplement: Supplementary file 1 [file Data_Sheet_1.docx]

**Tables**

**Table 1**

*Table of themes and references used in thematic map*

| Stage | Theme | Subtheme | Source |
| --- | --- | --- | --- |
| Pre-change | Organizational culture |  | Belisari et al. (2020) |
|  |  | Vision and goals | Taylor et al. (2015); Obal and Morgan (2018) |
|  |  | Strategy | Wang et al. (2015); Parris et al. (2016); Shin and Shin (2020) |
|  |  | Values and norms | Ford et al. (2016); Tamayo-Torres et al. (2016); Tseng (2017); Han et al. (2020) |
|  | Organizational structure | Power structure and work routineness | Avadikyan et al. (2016); Wang and Feeney (2016) |
|  |  | Policies and regulations | Wells et al. (2015) |
|  | Leadership and management | Cognizance | Tamayo-Torres et al. (2016); Wang and Feeney (2016); Eze et al. (2019) |
|  |  | Leadership style | Yahaya and Ebrahim (2016); Tseng (2017); Farahnak et al. (2020); Sony and Naik (2020) |
|  |  | Participative management | Vrhovec et al. (2015); Andersen (2016); Martins et al. (2016); Zhang and Xiao (2017); Maali et al. (2020) |
|  |  | Sensemaking management amongst multiple stakeholders | Costa et al. (2014); Jiao and Zhao (2014); Taylor et al. (2015); Andersen (2016); Heath and Porter (2019) |
|  |  | Emerging influencers | Kim (2015); Taylor et al. (2015); Andersen (2016); Hao and Padman (2018) |
|  |  | Technology selection and human factor analysis | Sætren et al. (2016); Efremovski et al. (2018); El Hamdi and Abouabdellah (2018); Keyworth et al. (2018); Heath and Porter, (2019); Molino et al. (2020); Sony and Naik (2020) |
|  |  | Resource management and reconfiguration | Pace (2016); Ali et al. (2018) |
|  | Resources | External resources | Antonelli and Scellato (2013); Pace (2016) |
|  |  | Internal resources | Bayerl et al. (2013); Vella et al. (2013); Nielsen and Mengiste (2014); Sætren and Laumann (2014); Freeze and Schmidt (2015); Huang (2015); Naor et al. (2015); Vrhovec et al. (2015); Bala and Venkatesh (2016); Langstrand (2016); Zhang and Xiao (2017); Vaishnavi et al. (2019); Gillani et al. (2020) |
|  |  |  |  |
| Change process | Organizational intervention | Communication about the technology change | Kierkegaard (2015) |
|  |  | Participation of key actors and users in technology adoption | Jensen and Kushniruk (2014); Rizzuto et al. (2014); Johannsdottir et al. (2015) |
|  |  | Training for key adopters and mediators | Petit dit Dariel et al. (2013); Takian et al. (2014); Escobar-Rodríguez and Bartual-Sopena (2015); Herbert and Connors (2016); Shirish et al. (2016); Mühlburger et al. (2017); Keyworth et al. (2018); Kumar et al. (2018); Molino et al. (2020); Thomas and Yao (2020) |
|  | Human-technology interaction | Technology sensemaking | Vella et al. (2013); Jiao and Zhao (2014); Bourrie et al. (2015); Escobar-Rodríguez and Bartual-Sopena (2015); Freeze and Schmidt (2015); Goldkind et al. (2016); Hopp and Gangadharbatla (2016); Martins et al. (2016); Nagy et al. (2016); Vest and Kash (2016); Singh (2017); Barrett (2018) |
|  |  | Physical proximity and accessibility | Vella et al. (2013) |
|  |  | Technical support | Freeze and Schmidt (2015); Wells et al. (2015); Avadikyan et al. (2016); Kumar et al. (2018); Han et al. (2020) |
| Post-change | Resistance |  | Freeze and Schmidt (2015); Klaus et al. (2015);  Andersen (2016); Barrett (2018); Cichosz et al. (2020); Obal and Morgan (2018); |
|  |  | Aggressive non-use |  |
|  |  | Workaround | Freeze and Schmidt (2015); Vrhovec et al. (2015) Choudrie and Zamani (2016); Heath and Porter (2019); Malaurent and Karanasios (2020) |
|  |  | Involuntary non-use | Andersen (2016) |
|  |  |  |  |
|  | Acceptance | Assimilation and use as intended | Sætren and Laumann (2014) |
|  |  | Customization and technological evolution | Wells et al. (2015); Ford et al. (2016); Malaurent and Karanasios (2020) |
| Present in all stages | External environment |  | Bayerl et al. (2013); Kapoor and Lee (2013); Poba-Nzaou et al. (2014); Aldossari and Mokhtar (2020); Han et al. (2020); Nielsen and Mengiste (2014); Bhuyan et al. (2014) |

**Table 2**

*An overview of the descriptive analysis of the publications used for the thematic map*

| Classification of references | Reference (%) |
| --- | --- |
| Sector |  |
| Management, business, and economics | 35 |
| Health | 31 |
| Information science and computer science | 18 |
| Education | 5 |
| Engineering | 5 |
| Public administration and law | 5 |
| Region |  |
| North America | 37 |
| Europe | 35 |
| Asia | 16 |
| Intercontinental | 8 |
| Australia | 3 |
| South America | 1 |
| Method |  |
| Qualitative | 45 |
| Quantitative | 41 |
| Other (Mixed method/Literature review) | 15 |

**Table 3**

*Themes and subthemes in thematic map for pre-change stage*

| Theme | Subtheme |
| --- | --- |
| Organizational culture | Vision and goal |
|  | Strategy |
|  | Values and norms |
| Organizational structure | Power structure and work routineness |
|  | Policies and regulations |
| Leadership and management | Cognizance |
|  | Leadership style |
|  | Participative management |
|  | Sensemaking management amongst multiple stakeholders |
|  | Emerging influencers |
|  | Technology selection and human factor analysis |
|  | Resource management and reconfiguration |
| Resources | External resources |
|  | Internal resources |
|  |  |
|  |  |

**Table 4**

*Themes and subthemes in thematic map for change stage*

| Theme | | Subtheme |
| --- | --- | --- |
| Organizational intervention | Communication about the technology change | |
|  | Participation of key actors and users in technology adoption | |
|  | Training for key adopters and mediators | |
| Human-technology interaction | Technology sensemaking | |
|  | Physical proximity and accessibility | |
|  | Technical support | |

**Table 5**

*Themes and subthemes in thematic map for post-change stage*

| Theme | Subtheme |
| --- | --- |
| Resistance | Aggressive non-use |
|  | Workaround |
|  | Involuntary non-use |
| Acceptance | Assimilation and use as intended |
|  | Customization and technological evolution |
